# Amyloid Formation by the Pro-Inflammatory S100A8/A9 Proteins in the Ageing Prostate

**DOI:** 10.1371/journal.pone.0005562

**Published:** 2009-05-15

**Authors:** Kiran Yanamandra, Oleg Alexeyev, Vladimir Zamotin, Vaibhav Srivastava, Andrei Shchukarev, Ann-Christin Brorsson, Gian Gaetano Tartaglia, Thomas Vogl, Rakez Kayed, Gunnar Wingsle, Jan Olsson, Christopher M. Dobson, Anders Bergh, Fredrik Elgh, Ludmilla A. Morozova-Roche

**Affiliations:** 1 Department of Medical Biochemistry and Biophysics, Umeå University, Umeå, Sweden; 2 Department of Medical Biosciences/Pathology, Umeå University, Umeå, Sweden; 3 Umeå Plant Science Center, Umeå University, Umeå, Sweden; 4 Department of Chemistry, Umeå University, Umeå, Sweden; 5 Department of Clinical Microbiology/Virology, Umeå University, Umeå, Sweden; 6 Department of Chemistry, University of Cambridge, Cambridge, United Kingdom; 7 Institute of Immunology, University of Münster, Münster, Germany; 8 Department of Neurology, University of Texas Medical Branch, Galveston, Texas, United States of America; 9 Department of Clinical Medicine, Örebro University, Örebro, Sweden; Tel Aviv University, Israel

## Abstract

**Background:**

The conversion of soluble peptides and proteins into polymeric amyloid structures is a hallmark of many age-related degenerative disorders, including Alzheimer's disease, type II diabetes and a variety of systemic amyloidoses. We report here that amyloid formation is linked to another major age-related phenomenon − prostate tissue remodelling in middle-aged and elderly men.

**Methodology/Principal Findings:**

By using multidisciplinary analysis of corpora amylacea inclusions in prostate glands of patients diagnosed with prostate cancer we have revealed that their major components are the amyloid forms of S100A8 and S100A9 proteins associated with numerous inflammatory conditions and types of cancer. In prostate protease rich environment the amyloids are stabilized by dystrophic calcification and lateral thickening. We have demonstrated that material closely resembling CA can be produced from S100A8/A9 *in vitro* under native and acidic conditions and shows the characters of amyloids. This process is facilitated by calcium or zinc, both of which are abundant in *ex vivo* inclusions. These observations were supported by computational analysis of the S100A8/A9 calcium-dependent aggregation propensity profiles. We found DNA and proteins from *Escherichia coli* in CA bodies, suggesting that their formation is likely to be associated with bacterial infection. CA inclusions were also accompanied by the activation of macrophages and by an increase in the concentration of S100A8/A9 in the surrounding tissues, indicating inflammatory reactions.

**Conclusions/Significance:**

These findings, taken together, suggest a link between bacterial infection, inflammation and amyloid deposition of pro-inflammatory proteins S100A8/A9 in the prostate gland, such that a self-perpetuating cycle can be triggered and may increase the risk of malignancy in the ageing prostate. The results provide strong support for the prediction that the generic ability of polypeptide chains to convert into amyloids could lead to their involvement in an increasing number of otherwise apparently unrelated diseases, particularly those associated with ageing.

## Introduction

The reproductive role of the prostate gland decreases with increasing age, leading to prostate tissue remodelling. This can be accompanied by serious problems such as benign prostatic hyperplasia, observed in 70% of men in their 60 s [1], and prostate cancer [2]. The latter is the most common non-cutaneous malignant neoplasm in men in Western countries [2]. The incidence of prostate cancer is rising rapidly with ageing population and now affects several millions men in Western world. In USA alone ca. 190 000 new cases are reported yearly and ca. 29 000 deaths have occurred from prostate cancer in 2008 according to the surveillance of the National Cancer Institute. In Europe mortality rates from prostate cancer varies significantly among different countries [3], [4]. There is a marked contrast between Mediterranean regions with below-average mortality and the other states, where there are several canters of excess mortality, including Sweden, Denmark, West of Germany, North of France, Ireland and Netherlands. There is a lack of understanding of the factors which may affect the increasing incidence of disease and its obvious geographic pattern. It appears that prostate pathologies may be a cost of longevity in the post-reproductive period. In order to provide an insight into the potential causes of prostate pathologies, we have carried out systematic and multidisciplinary studies of common prostate inclusions denoted as corpora amylacea [4], which are found in a significant proportion of males over the age of 50.

There is a growing body of evidence indicating that inflammation plays a crucial role in prostate pathogenesis, as it is found to be associated with 40–90% of benign prostatic hyperplasia [1] as well as with 20% of all human cancers [2], [5]. CAs are thought to be linked clinically to asymptomatic prostate inflammation and are often observed adjacent to the damaged epithelium and focal inflammatory infiltrates [2], [6], [7]. CAs have been also detected in 55% of cases in a study of high-grade prostatic intraepithelial neoplasia in specimens derived from radical prostatectomies [8]. The prostate CA depositions are often of a few millimetres in diameter, but their bulk weight can in some instances constitute up to a third of the weight of the prostate gland. The inclusions bodies coined as CA have been also found in the brain [9], lung [10], ovary [11] and uterus [10]. Their incidence is commonly associated with ageing and they may be of a very diversified origin [9]–[13]. Brain CAs have been observed much more frequently in patients suffering from Alzheimer's disease and other neurodegenerative conditions rather then in normal ageing [9], [14], [15]. Indeed, it has been suggested that in the development of CA in the brain, the initiating process is most probably degenerative in nature, following the synthesis of stress proteins [13], [16].

Despite the high prevalence of the prostate CAs in later life [17], this is still a highly disputed area with regard to their nature and pathological significance in normal ageing and in prostate pathologies resulting from benign or malignant changes. In several early studies it was reported that prostatic CAs could contain amyloid structures [17]–[19]; however CAs were also viewed as calcified bodies, prostatic concretion or calculi, resulting from calcification of precipitated prostatic secretion [20], [21] or arising from simple precipitation of salts normally presented in prostatic fluid [22]. Localized amyloid deposits, which were not defined as CA, have been also described in prostate, seminal vesicles and in the lower urinary tract in some case studies on human patients by using clinical, radiological, MRI and immunohistopathological techniques [6], [23], [24]. The protein content of some prostate CA inclusions was investigated by immunohistochemical staining using a panel of antibodies against the major known in 1990s amyloidogenic proteins [10], [17]; β_2_-microglobulin was identified, but the antibodies to other proteins, which were not known as amyloidogenic thus far, have not been subjected to this examination. Besides this, the application of immunostaining as a sole method to detect proteins proved to be unreliable, especially if potential candidates may be present not in the native state, but in their amyloid form [25].

In the present work we have characterized CA inclusions extracted from seven radical prostatectomy specimens from human patients diagnosed with prostate cancer. By using a range of modern biochemical and biophysical techniques we have shown that the pro-inflammatory calcium-binding proteins S100A8 and S100A9, also known as calgranulin A and calgranulin B, respectively, play an important role in their formation. The S100A8 and S100A9 belong to a multigenic and multifunctional family of about 20 members of calcium-binding S100 proteins [26], [27]. A heterodimer of S100A8/A9 has emerged as an important pro-inflammatory mediator in acute and chronic inflammation [28]–[30]. Increased levels of S100A8 and S100A9 have been detected in various human cancers, being abundantly expressed in neoplastic cells and also in infiltrating immune cells [29], [31]–[33]. In particular, the enhanced secretion of S100A8 and S100A9 was found in human prostate cancer cells [34], [35]. Altogether, their expression patterns, potential cytokine-like functions, up-regulation and regulation via signalling pathways, including tumor-promoting RAGE receptor, suggest that S100A8/A9 may play a key role in inflammation-associated cancers [35], [36]. Moreover, emerging evidence revealed a general role for the S100A9 protein in the phenomenon of dystrophic calcification, specifically in calcifying matrix vesicles and atherosclerotic plaques in the arterial wall [37]. It is interesting to note, that nine out of ten immunohistochemically analyzed S100 proteins of the S100 family were also found in CA deposits in normal human brain tissue [9]. The S100A8 and S100A9 molecules are characterized by a conformational variability, adopting hetero and homo-dimeric, trimeric as well as tetrameric complexes, that can also contribute to their multiple functionality [38]–[41]. Although many functions have been identified and proposed for S100A8/A9, their roles in various biological processes still remain to be defined. For the first time we report that S100A8/A9 proteins readily form amyloid structures both *in vivo* and *in vitro* and they are present in the prostate CA inclusions in their amyloid form.

## Results

### Protein identification in CA inclusions by mass-spectrometry

In order to identify the specific proteins within CA, the samples were solubilized in ice cold TE buffer and analyzed by liquid chromatography coupled with electrospray ionization mass-spectrometry (Table 1). In all the specimens examined the data reveal the presence of 3 major proteins, S100A8, S100A9 and human serum albumin. A several auxiliary and bacterial proteins were also identified (Table 1 and 2), but these proteins varied substantially from patient to patient and their quantities were too low to be detected by subsequent gel electrophoresis with silver staining.

**Table 1:** Proteins identified in CA samples from the selected group of patients by liquid chromatography-electrospray ionization mass spectrometry.

Protein	Accession number	‡Protein size (kDa)	Number of identified unique peptides (‡sequence coverage %) in following patients (P).						
			P1	P2	P3	P4	P5	P6	P7
S100A8	IPI00007047	10	2 (24)	1 (11)	1 (11)	2 (24)	2 (24)	3 (31)	3 (31)
S100A9	IPI00027462	13	3 (36)	2 (25)	1 (13)	1 (13)	1 (13)	4 (31)	3 (26)
Human serum albumin	IPI00745872	67	10 (24)	6 (14)	24 (60)	16 (38)	13 (31)	#	5 (10)
α-1-acid glycoprotein 1	IPI00022429	21	#	1 (8)	2 (13)	2 (24)	1 (8)	#	#
Zinc-α-2-glycoprotein	IPI00166729	32	#	#	3 (16)	5 (21)	3 (12)	#	#
Hemoglobin subunit α	IPI00410714	15	2 (14)	#	1 (11)	#	#	1 (11)	#
Hemoglobin subunit β	IPI00654755	16	1 (9)	#	#	#	#	#	#
Cathepsin G	IPI00028064	27	2 (9)	#	#	#	#	3 (11)	#
Neutrophil defensin 1	IPI00005721	8	1 (12)	#	#	#	#	#	#
Myeloperoxidase	IPI00007244	79	1 (2)	#	#	#	#	12 (10)	#
Prostate-specific antigen	IPI00010858	27	#	#	#	#	1 (5)	#	#
Haptoglobin	IPI00431645	31	#	#	5 (25)	#	#	#	#

‡ Calculated after removing signal peptide from their precursor sequence. # No significant identification.

**Table 2:** *Escherichia coli* (*E. coli*) proteins identified in CA samples by liquid chromatography-electrospray ionization mass spectrometry.

Protein	Accession number	Organism	Protein size (kDa)
Co-chaperonin GroES	NP_290775	*E. coli*	10
Heat shock protein GrpE	NP_289166	*E. coli*	22
ATP-dependent protease regulatory subunit	CAA40846	*E. coli*	66

A single peptide from each protein was identified.

### Presence of bacteria in CA inclusions

The CA samples from 5 patients were further analyzed for the presence of bacterial DNA by the polymerase chain reaction (PCR) method [42]. Bacterial 16s rDNA was detected in all specimens (Figure S1). The PCR products were cloned and 5 clones from each specimen were sequenced. Altogether, 13 distinct sequences (323–338 bps) were identified, 8 of which shared >98% homology with *Escherichia coli* sequences, 3 corresponded to uncultured bacterial clones and 2 showed <98% homology to published sequences. 4 out of 5 *Escherichia coli* rDNA positive samples have been shown also to contain bacterial proteins (Table 2).

### Amyloid nature of the major proteinaceous components of CA

**Figure 1:**
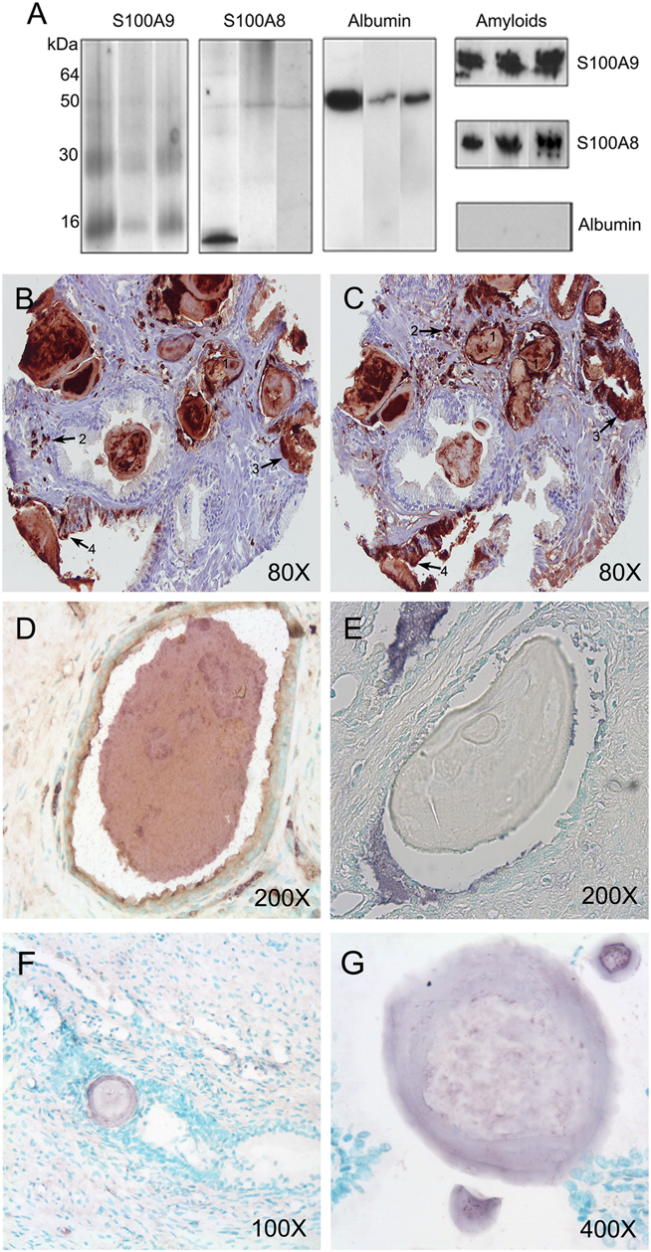
Immunoassays of CA proteinaceous components. (A) Western blots of CA extracts from three representative specimens with S100A9, S100A8 and human serum albumin antibodies; the staining of aggregates in stacking gel with corresponding antibodies is shown in the right column. (B and C) Immunostaining of CA and surrounding tissues with S100A8 and S100A9 antibodies, respectively, shown in brown; the positive S100A8/A9-staining of CA (1), macrophages in stroma (2), macrophages in epithelium tissues (3) and the focal epithelial staining (4) are indicated by numbers and arrows. (D) Co-immunostaining of CA inclusions with anti-S100A8 (shown in purple) and anti-S100A9 antibodies (shown in brown). (E) Lack of CA immunostaining by human serum albumin antibodies (CA body does not display purple colour). (F and G) Immunostaining of the prostate CA inclusions by antibodies towards amyloid fibrils shown in purple with low and high magnification. The magnification is indicated in left lower corner of each image.

The extracts from the CA samples were subjected to SDS-PAGE electrophoresis and then to Western blot analysis with polyclonal antibodies towards S100A8, S100A9 and human serum albumin (Figure 1A). The S100A9 antibodies recognized 14 kDa monomeric and 28 kDa dimeric species [38]–[40], the S100A8 antibodies recognized 10 kDa monomeric S100A8 (Figure 1A) in 3 out of 7 specimens analyzed. Both the S100A8 and S100A9 antibodies recognized a 48 kDa species, suggesting the presence of hetero-tetramers of S100A8/A9 [38]–[40]. Human serum albumin antibodies interacted with a ca. 50 kDa molecular species, indicating the presence of truncated serum albumin (the molecular weight of the intact protein is 67 kDa). However, aggregated species with a high molecular weight, and which remained in the stacking gels, did not interact with anti-serum albumin antibodies. They were stained by both S100A8 and S100A9 antibodies, indicating that these aggregates are composed of both these proteins (Figure 1A).

The CA samples were also subjected to immunohistochemical analysis, which revealed that they are stained positively with both S100A8 and S100A9 antibodies (Figure 1B–1D). In the tissues adjacent to CA inclusions we observed positive foci of S100A8 and S100A9, including the S100A8 and S100A9 positively stained glandular epithelial cells and tissue macrophages, which infiltrate inflamed glands (Figure 1B and 1C). The co-immunostaining of CA inclusions with both antibodies towards S100A8 and S100A9 proteins is shown in Figure 1D with higher magnification. By contrast, only weak staining with serum albumin antibodies was observed at the edges of the CA inclusions and in the surrounding tissues (Figure 1E), indicating that serum albumin, detected by mass-spectrometry and Western blot analysis, came from the surrounding tissues, and not from the CA bodies. All CA specimens were also stained with anti-amyloid fibril antibodies [43] (Figure 1F and 1G) and with Congo red dye, used as a marker for the presence of the amyloid form of proteins (Figure S2). These results demonstrate that the amyloid material constitutes a significant mass of the CA specimens.

### Structural characterisation of S100A8/A9 amyloid formation

**Figure 2:**
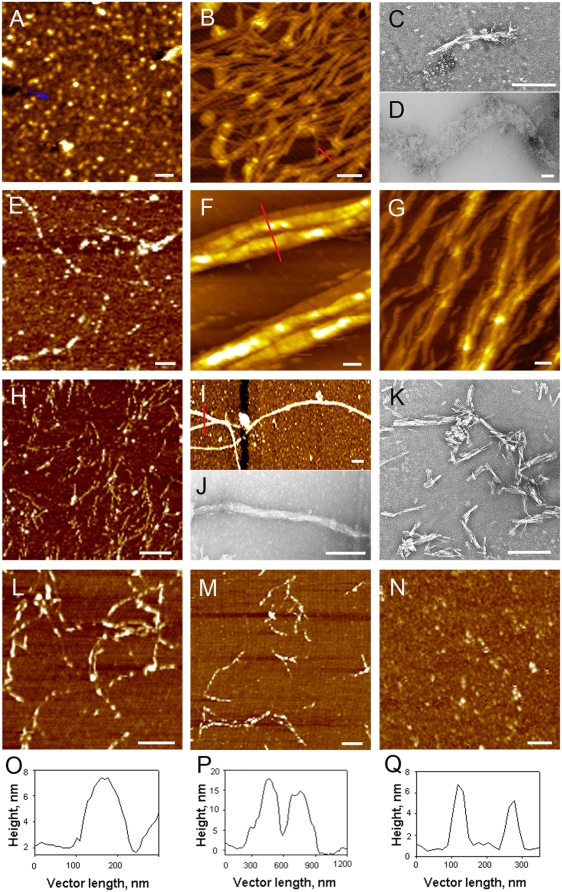
AFM and TEM imaging of *ex vivo* and *in vitro* S100A8/A9 amyloid structures. (A) AFM images of *ex vivo* amyloid oligomers, chain-like sequences are indicated by blue arrow; (B) *ex vivo* amyloid fibrillar network; (C,D) TEM images of *ex vivo* amyloid superstructures; (E) AFM images of S100A8/A9 oligomeric assemblies produced at pH 7.4, 37°C with agitation after 2 week of incubation, (F,G) fibrillar bundles produced at pH 7.4, 37°C with agitation after 8 weeks; (H–K) heterogeneous types of S100A8/A9 amyloids produced at pH 2.0 and 57°C after 4 weeks of incubation with residual content of Ca and Zn. S100A8/A9 amyloids produced at pH 2.0 and 57°C after 2 weeks of incubation in the presence of (L) 1 mg/ml powdered Ca_3_(PO4)_2_ and (M) 10 mM ZnCl_2_; (N) aggregates observed in the presence of 50 mM EDTA. AFM cross-section analysis of amyloid structures indicated by red lines in corresponding images (O from B; P from F and Q from I). Scale bars are shown in white and equal to 250 nm in all AFM images.

Atomic force microscopy (AFM) and transmission electron (TEM) microscopy were used to examine the *ex vivo* CA extracts, and they revealed the presence of a variety of highly heterogeneous aggregates (Figure 2). These aggregates include spherical species of ca. 2–3 nm in height (Figure 2A), some of them arranged into chain-like sequences, which were closely similar to the oligomeric precursors of amyloid fibrils described for numerous peptides and proteins [44]–[46]. Extensive networks of fibrillar species of 4–8 nm in height and several microns in length were observed (Figure 2B and 2O), characteristic of mature amyloid fibrils. Assemblies of straight and rigid fibrils a few hundred nanometres in length (Figure 2C) were present in all CA specimens, as well as there were larger scale super-molecular assemblies, reaching a few microns in length and up to ca. 500 nm in thickness (Figure 2D).

In order to examine further the amyloidogenic properties of the S100A8/A9 proteins we set out to produce their amyloid forms *in vitro*. The S100A8/A9 complexes, extracted from granulocytes and produced recombinantly from *Escherichia coli*, were each incubated under the native conditions of pH 7.4 and 37°C with agitation and at pH 2.0 and 57°C without agitation. The aliquots were collected regularly for further examination during the period of 2 months; the sub-millimolar concentrations of metal ions were present in the samples after the purification procedure [40], [47]. Under both conditions the proteins were found to assemble into heterogeneous fibrillar species. At pH 7.4, species resembling *ex vivo* oligomers and short protofilaments were observed after 2 weeks of incubation (Figure 2E). Upon further incubation for up to 8 weeks, thick bundles of fibrils with heights of 15–20 nm and a few microns in length constituted the major population of fibrillar aggregates (Figure 2F, 2G and 2P).

In the S100A8/A9 samples incubated at pH 2.0 without agitation the spherical species and protofilaments also emerged in 2 weeks (Figure 2H), and after 4 weeks the flexible fibrils with height of ca. 4–5 nm and microns in length (Figure 2I and 2Q) were observed together with straight and rigid fibrillar structures a few hundred nanometres in length (Figure 2K), which resembled the *ex vivo* species shown in Figure 2C. Large fibrillar bundles, reaching ca. 80 nm in diameter (Figure 2J) and resembling the superstructures shown in Figure 2D, were also regularly found in specimens incubated for 4–6 weeks.

As *ex vivo* CA deposits were found to be calcified and to contain zinc salts, we examined the effect of these ions on amyloid formation by S100A8/A9. The S100A8/A9 amyloid protofilaments of ca. 2 nm height were assembled in the presence of ZnCl_2_ and in a suspension of Ca_3_(PO4)_2_ (Figure 2L and 2M), but not when EDTA was added in solution even during 2 weeks of incubation (Figure 2N). These species converted into the fibrillar assemblies described above upon prolonged incubation, and again no filamentous structures developed in the presence of EDTA.

### Aggregation propensity profiles of S100A8/A9

**Figure 3:**
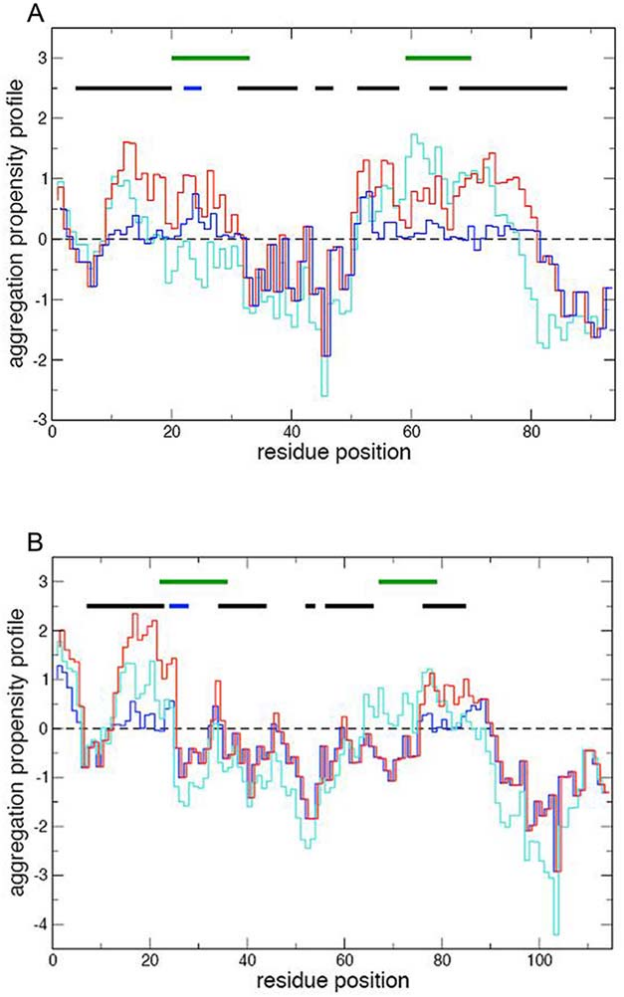
Intrinsic aggregation propensity profiles. (A) S100A8 and (B) S100A9 aggregation propensity profiles denoted at pH 7.0 by *red*, at pH 2.0 – by *turquoise* and in the natively folded S100A8/A9 complex – by *blue lines*, respectively. Calcium-binding sites are indicated by *green*, α-helices – by *black* and β-strands – by *blue bars*, respectively.

The identification of the regions of peptide and protein sequences, that are likely to be most important in promoting amyloid formation, enables us to predict the behaviour of these two proteins under given conditions. Intrinsic aggregation propensity profiles [48], [49] of monomeric S100A8 and S100A9 at pH 7.0 and pH 2.0, the conditions which we have used for *in vitro* amyloid formation, and in their natively folded S100A8/A9 oligomeric complex were calculated (Figure 3). The overall aggregation scores for S100A8 are 0.76 at pH 7.0 and 0.77 at pH 2.0 and for S100A9, 1.04 and 0.65, respectively; the aggregation score of S100A9 in particular is similar to the aggregation scores of Aβ(1–40) and Aβ(1–42) peptides at pH 7.0, which are equal to 0.97 and 0.94, respectively [48]. In the S100A8/A9 oligomeric complex (references for the structures: pdb 1xk4 for S100A8 and pdb 1irj for S100A9) the aggregation profiles (Figure 3) and the scores of 0.18 and 0.32 for S100A8 and S100A9, respectively, are significantly reduced, indicating that most of the aggregation-prone sequences are involved in the oligomeric interactions. In both proteins the calcium-binding sites with low affinity (amino acid residues 20–33 for S100A8 and 23–36 for S100A9) and high affinity (amino acid residues 59–70 for S100A8 and 67–78 for S100A9) are located in close proximity to the segments that are highly aggregation-prone. As S100A8 and S100A9 at neutral pH undergo a calcium-dependent oligomerization rather than amyloid formation [39] and as the intermolecular interactions responsible for native state oligomerization and aggregation can overlap, calcium-binding is likely to generate conformational changes that, in addition to promoting native state oligomerization, increase the aggregation process as demonstrated above. Hence, we surmise that the evolution of a calcium-dependent oligomerization required to generate the native functional state, has come at the cost of a higher calcium-dependent aggregation propensity.

### Analysis of the mineral phases of CA

**Figure 4:**
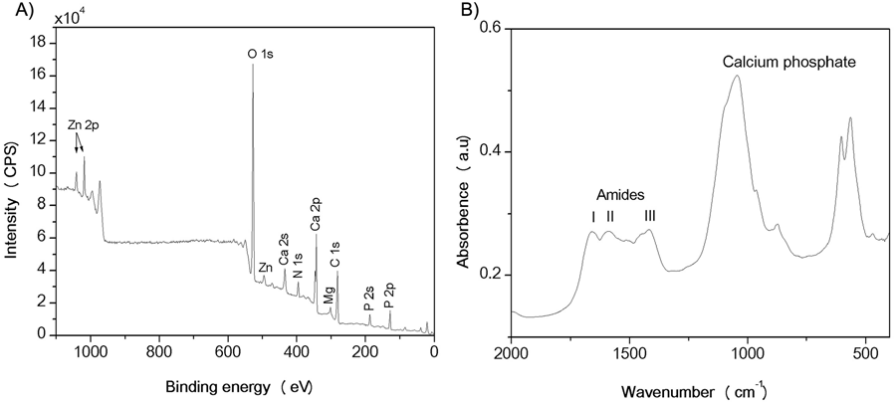
Chemical content of CA inclusions. (A) XPS survey and (B) FTIR spectra of powdered prostate CA material.

**Figure 5:**
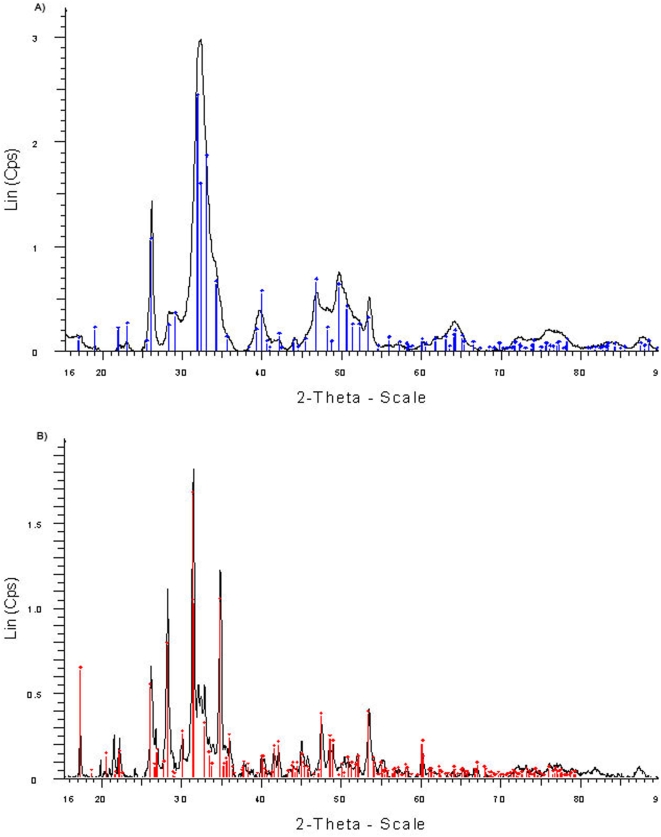
X-ray powder diffraction patterns of CA inclusions. (A) Hydroxylapatite − (Ca_5_(PO_4_)_3_OH) and (B) whitlockite − (Ca_2_(PO4)_3_) crystalline phases of CA.

In order to determine the mineral components of CA inclusions, the powdered CA samples were subjected to x-ray photoelectron spectroscopy (XPS) analysis. A typical XPS spectrum of CA (Figure 4A) reveals the presence of C, N, O, P, Ca, K, Zn and Mg atoms. The binding energies derived from the Ca 2p_3/2_ (347.4 eV) and P 2p_3/2_ (133.2 eV) lines and the Ca/P atomic ratio (1.3∶1.0±0.2) indicate the presence of calcium phosphate phases as a major inorganic component of CAs. The existence of hydroxylapatite (Ca_5_(PO4)_3_OH) (Figure 5A) and whitlockite (Ca_2_(PO4)_3_) (Figure 5B) crystalline phases in various CA samples was also confirmed by x-ray powder diffraction measurement. The atomic concentrations of Zn (ca. 0.3 atomic %) and Mg (1.5–2.0 atomic %) indicate that these ions are incorporated into the calcium phosphate phases to form crystalline compounds, in which up to 20% of Ca cations are substituted by Zn or Mg. The atomic concentrations and binding energies corresponding to the photoelectron lines of N 1 s (NH, 400.0 eV, atomic concentration 5–6%), C 1 s (C−(C,H), 285.0 eV, atomic concentration ca. 13.5%; C−(O,N), 286.4 eV, atomic concentration ca. 5.7%; COOH, 288.1 eV, atomic concentration ca. 5.6%) and O 1 s (532.5 eV, atomic concentration ca. 6.9%) indicate that the remaining component of CA is proteinaceous in nature (NIST Standard Reference Database 20). A sample of CA was also probed at 350°C in an ashing furnace, which resulted in a 32% weight loss; this observation indicates that 30–40% of CA is organic in nature. XPS spectra recorded for the CA sample from different patients showed that the variation in the weight of mineral and organic compounds was within 10–20% and the variations in Zn content were within 0.1–0.5 atomic %. Consistent with the XPS analysis, the Fourier transform infrared spectrum of CA powder shows characteristic amide I–III bands of 1700–1359 cm^−1^, corresponding to peptide bonds in the proteinaceous phase, and the broad bands at 1100–900 cm^−1^ and 650–500 cm^−1^, which can be attributed to the vibration mode of the phosphate group in calcium phosphate (Figure 4B).

## Discussion

The results described in this study demonstrate that the prostate CA inclusions are primarily composed of calcified amyloid forms of the pro-inflammatory S100A8/A9 proteins. As prostatic fluid is very rich in protein content [50], [51], it is not unexpected that small quantities of a range of other proteins were also found in the CA inclusions, presumably being trapped in the aggregating and growing deposits (Table 1 and 2). We have not detected β_2_-microglobulin reported previously in CA deposits by immunohistochemical analysis [17], even through we were able to detect small traces of bacterial proteins due to the high sensitivity of the liquid chromatography-electrospray ionization mass spectrometry technique, utilized in this study. We have investigated here the CA inclusions from the Scandinavian population; although probability cannot be excluded that prostate CA deposits can be diversified in their origin, depending on ethnic and geographical factors as well as their clinical history. Extended epidemiological studies on the male populations from different geographical and climate zones would be of significance in addressing this issue as well as in revealing the causative factors affecting the geographic pattern of prostate cancer incidence discussed above [3], [4].

**Figure 6:**
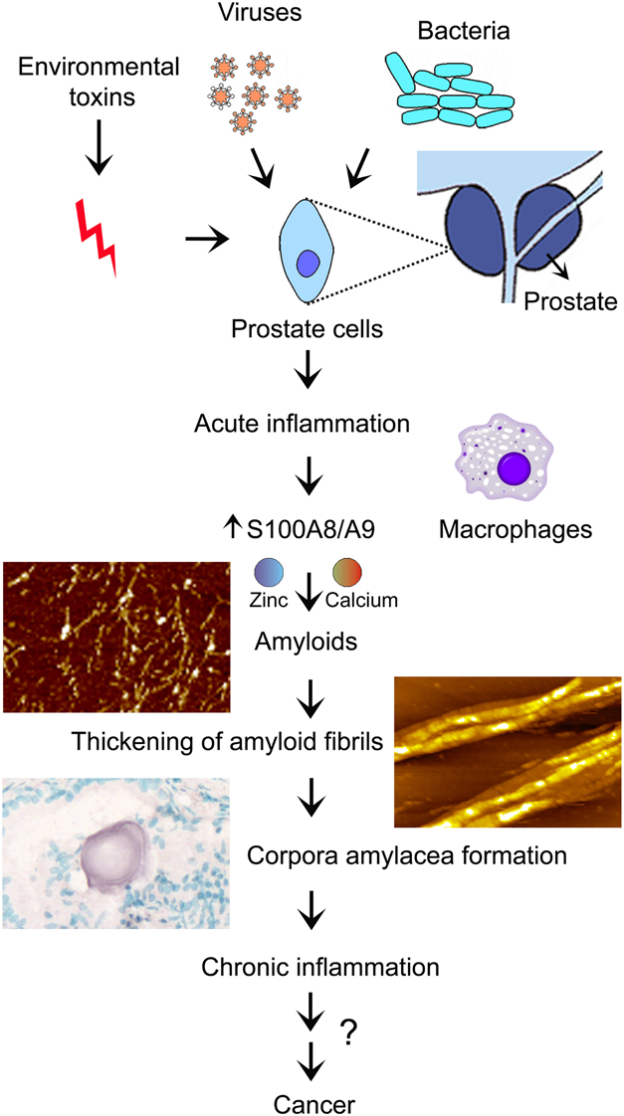
Inflammation-dependent pathways of the prostate CA formation. Infection, inflammation and amyloid self-assembly of S100A8/A9 proteins in the presence of calcium or/and zinc are important factors leading to CA depositions in the prostate gland.

In inflamed tissues adjacent to the CA inclusions we have commonly observed S100A8 and S100A9 positive focal epithelial cells and macrophages (Figure 1B and 1C). These activated cells may contribute to the rise in the concentrations of S100A8 and S100A9 at the sites of inflammation. As amyloid formation is a concentration-dependent process, the increasing concentration of aggregation-prone proteins in the sites of inflammation would favour their amyloid assembly and deposition in the CA (Figure 6).

The finding of *Escherichia coli* DNA (Figure S1) and *Escherichia coli* proteins (Table 2), in the CA deposits makes it possible to hypothesize that CA formation may be associated with bacterial infection, taking place in the prostate during the prolonged course of their initiation and growth. Indeed, we and others have detected *Escherichia coli* in the prostate tissues [52]–[54] Therefore the CA, entrapping biological molecules such as *Escherichia coli* DNA and proteins, may represent a snapshot of the prostate history with regards to bacterial infection affecting the prostate gland during their formation. As a CA grows and the inflammatory process spreads, neighbouring acini are obstructed and the cycle can continue.

Other amyloid diseases are known to be associated with chronic inflammation, including Alzheimer's and Parkinson's diseases, although it is far from clear, which role multifunctional inflammatory mediators play in different pathophysiological situations [55]–[59]. Growing evidence indicates that an abnormal deposition of fibrillar Aβ peptide in the brain can be accompanied by inflammatory responses and increasing production of pro-inflammatory cytokines in microglia. Amyloid formation by the pro-inflammatory S100A8/A9 proteins, their deposition in the ageing prostate and their association with local inflammatory reactions, all emphasise the role of persistent inflammation in the prostate pathogenesis. Growing CA deposits may induce further degradation of epithelial tissue in the prostate gland, exacerbating inflammation and, therefore, potentially contributing to neoplastic transformation [2], [5], [7]. Studies on a larger number of prostate samples, including both neoplastic and non-cancerous prostate, would further clarify this issue and should generate a link between the pathological cascade including infection, inflammatory response and amyloid deposition with benign and malignant transformations in the prostate gland.

The identification of bacterial DNA and proteins in CA, including the highly amyloidogenic co-chaperonin GroES [60], can be related not only to the fact that bacterial infection is a contributory factor to inflammation, but suggests the potential role of bacterial infection in amyloid depositions (Figure 6). Currently, the link between amyloid formation and bacterial and viral infections receives growing attention [61]–[63]. In particular, it has been shown that semen-derived amyloid fibrils of the fragments of prostatic acidic phosphatase drastically enhance HIV infection in humans [64]. It would be important to examine whether the amyloid structures of S100A8/A9 proteins in prostate may act as a seed, promoting the amyloid formation of prostatic acidic phosphatase fragments in prostatic fluid. It is not excluded that the amyloid structures of bacterial origin such as amyloidogenic chaperonin GroES may also contribute to amyloid seeding of S100A8/A9 proteins themselves. Indeed, it has been demonstrated that curli from *Escherichia coli* and Sup35 from *Saccharomyces cerevisiae* can exert amyloid-accelerating properties in the murine serum AA amyloidosis [65]. The biochemical link between viral infection and the development of the Alzheimer's disease pathological features has also been examined [62], [63] and the authors suggested that herpes simplex virus type 1 can directly contribute to the development of senile plaques. While the amyloid fibrils in bacteria, fungi insects and in *de novo* design were found to fulfil useful functions [66], [67], amyloidogenesis in general still requires tight regulation to avoid amyloid toxicity. In most cases amyloid fibrils are detrimental in the host, leading to pathologies. Due to their high potency in seeding and cross-seeding, the misfolding of proteins into an amyloid state can be a general origin of infectivity, which was highlighted in the phenomenon of amyloid formation and propagation by prion proteins [68].

The hetero-oligomeric complexes of S100A8/A9 are characterised by significant stability and protease resistance comparable to these of prions [41]. In the protease rich environment of prostate gland, and especially at sites of inflammation, proteases are present at even higher levels than in other tissues. Protease resistance of the S100A8/A9 proteins could favour their accumulation and conversion into amyloid structures in prostate tissue. The bundles of amyloid fibrils of S100A8/A9 proteins, that are formed both *in vivo* and *in vitro* (Figure 2), are amongst the largest super-molecular species reported for amyloid assemblies [69]. The lateral association or thickening of the fibrils is likely to be a contributory factor to their stability in the prostate gland. Indeed, it has been suggested that the various functions of the S100A8/A9 hetero and homo-oligomers may be regulated by their differential protease sensitivity [41]. If so, the amyloid structures formed by the S100A8/A9 will be at the extreme end of the scale of resistance to proteolysis.

A recently reported function of S100A9 is associated with the promoting calcification [37], suggesting that this protein may also play a role in dystrophic calcification of CA deposits. The mineral content of CA was rather uniform in all the patients we studied (Figure 4A), suggesting that calcification is a regulated process and therefore could be influenced by the activities of S100A8/A9. In our *in vitro* studies we have shown that the formation of the amyloid structures of the S100A8/A9 proteins is promoted by the presence of calcium and zinc. The experimental observations were also supported by computational analysis of aggregation propensity profiles, demonstrating that the aggregation-prone regions are located in close proximity to the calcium- binding sites in both proteins. In the case of the S100A8/A9 proteins calcium-binding affects the competitive processes of protein folding and aggregation, while proteins themselves influence the process of calcification, which together may produce a synergistic effect, leading to the further enlargement of CA deposits (Figure 6).

Thus, the direct involvement of pro-inflammatory S100A8/A9 proteins in CA biogenesis, which we reported here for the first time, emphasizes their role in the age-dependent prostate remodelling and accompanied ailments (Figure 6). The discovery of the link between amyloid formation and prostate pathogenesis supports the paradigm that the generic ability of polypeptides to assembly into this stable form as an alternative to the native state [70], [71] is likely to result in its involvement in an increasing number of age-related diseases. The disorders as apparently unrelated as Alzheimer's and Parkinson's diseases and prostate pathologies can all be linked by the common phenomenon of amyloid formation. In all these diversified diseases inflammation can be an important causative factor in amyloid depositions. This conclusion gives further support to the strategies aiming at averting inflammation in order to treat and possibly prevent amyloid formation and also potential neoplastic developments in prostate.

## Materials and Methods

### CA specimens

CA specimens were dissected from the prostate tissue from seven patients undergoing radical prostatectomy due to prostate cancer. All patients gave their written informed consent and the collection of human tissues was approved by the Umeå research ethics board. The specimens were washed in sterile PBS and stored at −80°C.

### Protein samples

Human S100A8/A9 proteins were isolated from granulocytes as a heterodimer and stored in 20 mM Tris, 1 mM DTT, pH 7.2 [47]. Recombinant human S100A8 and S100A9 were expressed in *Escherichia coli*, their complex was produced as described previously [40] and they were kept in 20 mM Hepes, 140 mM NaCl, pH 7.4. The amyloid was produced by incubation of both purified from granulocytes S100A8/A9 complexes at 3.5 mg /ml and recombinant S100A8/A9 complexes at 2.3 mg/ml in 50 mM HCl, pH 2.0, 57°C without agitation and in the original buffers at pH 7.2 (pH 7.4), 37°C with agitation. All reagents were purchased from Sigma, unless stated otherwise.

### CA extractions

CA specimens were incubated in undiluted methanol at 4°C overnight, washed 3 times and ground in a glass homogenizer, adding ice cold TE buffer (20 mM Tris-HCl, 10 mM EDTA, pH 7.5) [72]. The homogenized material was centrifuged at 15000 g in a mini-centrifuge (Eppendorf). The pellet was re-suspended 3–4 times in TE buffer, homogenized through a 21gauge needle of 2 ml syringe and centrifuged again. TE buffer was sterilized and only sterile needles and syringes were used during the extraction procedures. All supernatants containing proteins were collected and stored at −20°C.

### CA extract tryptic digestion and peptide analysis by liquid chromatography-electrospray ionization mass spectrometry

CA extracts in 0.2 M NH_4_HCO_3_, 15 mM DTT were incubated at 95°C for 15 min, cooled to room temperature, mixed with 8 M urea and incubated for 1 h. Subsequently, the alkylation reaction was carried out at 37°C for 30 min in darkness in the presence of 80 mM iodoacetamide. Urea concentration was reduced to 0.8 M by diluting with 0.2 M NH_4_HCO_3_. Trypsin was added at 1∶40 enzyme-to-substrate ratio and tryptic digestion was carried out overnight at 37°C. It was stopped by adding formic acid to the final concentration of 0.5%. The resulting peptides were lyophilized and re-suspended in 1% TFA. Then they were desalted by using a Poros 50 reverse-phase R2 material (PerSeptive Biosystems) prepared in a GELoader tip (Eppendorf) as described previously [73]. The peptides were eluted from the R2 micro-column by using 80% acetonitrile with 0.1% TFA, lyophilized and re-suspended in 0.1% formic acid.

Subsequently, the tryptic peptides were subjected to a reversed-phase ultra-performance nano ACQUITY UPLC™ system (Waters). Each peptide sample was concentrated in a C18 trap column with symmetry of 180 µm×20 mm, 5 µm and washed with 5% acetonitrile and 0.1% formic acid at 15 µl/min speed for 1 min. The samples eluted from the trap column were subjected to a C18 analytical column (75 µm×100 mm, 1.7 µm) at 600 nl/min flow speed, using 0.1% formic acid as a solvent A and 0.1% formic acid in acetonitrile as a solvent B in a gradient. The following gradients were applied: linear from 0 to 40% B in 25 min, linear from 40 to 80% B in 1 min, isocratic at 80% B in 1 min, linear from 80 to 5% B in 1 min and isocratic at 5% B for 7 min.

The eluting analytes were sprayed into a Q-Tof Ultima™ mass spectrometer (Waters) with the capillary voltage set to 2.6 kV and cone voltage to 40 V. The instrument was calibrated using MS/MS fragments of GluFib peptide (Sigma Aldrich) and the samples offset calibration was performed as described previously [74]. The acquisition of MS/MS spectra was performed with an automated data-directed switching between the MS and MS/MS modes using the instrument software (MassLynx V4.0 SP4). The three most abundant signals of a survey scan (400–1300 m/z range, 0.87 s scan time and 0.13 s inter-delay) were selected by charge state, and collision energy was applied accordingly for sequential MS/MS fragmentation scanning (50–2000 m/z range, 0.9 s scan time, 0.1 s inter-delay). ProteinLynx Global Server software (V2.2.5) was used to convert raw data to peak lists for database searching.

The negative control measurements with the ice cold TE buffer and 0.2 M NH_4_HCO_3_, 15 mM DTT buffer used for dissolving CA material were performed. These solutions were subjected to the same procedures as the CA samples to exclude the general background level of contaminations.

### Protein identification by database analysis

Proteins were identified by a local version of Mascot search program V2.1.04 and Mascot Daemon application V2.1.6 (Matrix Science Limited, http://www.matrixscience.com), using human sequence library in the International Protein Index (IPI) database (IPI_human_20080409, 72,340 sequences). The following settings were used for the database search: trypsin-specific digestion with one missed cleavage allowed, carbamidomethylated cysteine set as fixed modification, oxidized methionine and deamidation in variable mode, peptide tolerance of 60 ppm and fragment tolerance of 0.1 Da. Peptides with Mascot ion scores exceeding the threshold for statistical significance of p<0.05 were selected and also re-processed manually to validate their significance.

### Western blot analysis

Gel electrophoreses were performed under reducing conditions by using 8–25% Phast gradient gels. Pre-stained molecular weight “SeeBlue” standards (Invitrogen) were included in each experiment. In Western blots, proteins were electro-transferred to nitrocellulose membranes by a Phast-system equipment (GE Healthcare). Non-specific reactivity was blocked by 5% non-fat milk in Tris-buffered saline, containing 0.05% Tween 20 (TBS-T) at 37°C for 1 h, washed 3×5 min with TBS-T and incubated with polyclonal rabbit anti-human S100A8, S100A9 (Santa Cruz) and serum albumin (Sigma) antibodies at 4°C overnight. The membranes were washed 3×5 min with TBS-T and incubated in the presence of horseradish peroxidase conjugated with anti-rabbit IgGs (GE Healthcare) at 1∶5000 dilution in TBS-T containing 5% non-fat milk at 37°C for 1 h. The blots were washed 3×15 min with TBS-T and the immuno-reactive proteins were detected by using the enhanced chemiluminescence kit (GE Healthcare).

### Immunohistochemistry

Archival prostate samples from patients with prostate cancer were dewaxed in xylene and dehydrated in ethanol series. After antigen retrieval in 10 mM Tris, pH 9.0 they were incubated with rabbit polyclonal anti-human S100A8, S100A9 (Santa Cruz) and serum albumin antibodies (Sigma). The CA samples were also stained with anti-amyloid fibril antibodies [43]. The immune reactivity was detected by an anti-rabbit IgG peroxidase Immpress reagent kit, followed by a Vector VIP or Vector DAB peroxidase substrate kits (Vector Laboratories Inc.). Negative controls were performed by substituting the primary antibodies with PBS or irrelevant antibody.

### 16s rDNA PCR and sequencing

CA aseptically removed from fresh prostatectomy samples were washed in sterile PBS prior to DNA extraction. DNA was purified by using E.Z.N.A. Blood DNA kit 11 (Omega Bio-TEK). 16s rDNA PCR and cloning were performed as described previously [42]. Plasmids were purified by using the QIAprep Spin miniprep kit (Qiagen) and sequenced by using the T3 primer (MWG Biotech AG). Database sequence matches to listed organisms were used to define a phenotype, if the closest match shared >98% similarity.

Sensitivity of the PCR corresponded to 200 colony forming units/reaction, which is significantly higher than the general background level. Stringent anti-contamination precautions to control PCR contamination were undertaken in the laboratory environment. The PCR laboratory space has complied or exceeded standard recommendations for PCR research, including isolated setup areas with dedicated pipettes, small reagent and primer aliquots, and use of aerosol-resistant pipette tips. The buffer solutions involved in the study were subjected to PCR analysis as negative controls to exclude potential reagent or laboratory contamination.

### Atomic force and electron microscopy

AFM measurements were performed on a PICO PLUS 5500 microscope (Agilent) as described previously [75]. The structural dimensions were determined by cross-section analysis in height images. TEM samples were applied to Formvar-coated nickel grids (400 mesh), stained with 2% uranyl acetate and viewed in a Philips CEM 100 (FEI) microscope. Fibrillar dimensions were obtained directly from the micrographs.

### Optical microscopy

The CA samples were stained with Congo red as described previously [76]. The samples were analysed under polarised light in an Olympus SZX10 stereo microscope (Olympus America Inc.) equipped with 20× GSWH20X eyepieces under 40−fold magnification.

### Protein aggregation propensity profiles

Intrinsic aggregation propensity profiles of S100A8/A9 proteins were calculated as described previously [48], [49] by using the pdb files of 1xk4 for S100A8 and 1irj for S100A9 from the Protein Data Bank.

### XPS spectrometry

XPS spectra were recorded using a Kratos Axis Ultra DLD electron spectrometer (Kratos Analytical) equipped with a monochromated Al K_α_ x-ray source operating at 150 W, a hybrid lens system including magnetic lenses and with a charge neutraliser [77]. The CA samples were instantly frozen and transferred to the analysis chamber with 2–4×10_−7_ Pa base-vacuum, −150°C. The wide-scan spectra (160 eV pass energy) and high resolution spectra of all elements (20 eV pass energy) were acquired. The binding energy scale was referenced to the C 1 s line of aliphatic carbon set at 285.0 eV. The spectra were processed by using a Kratos software. X-ray powder diffraction measurements were conducted on a D8 Advance powder diffractometer (Bruker).

### Fourier transform infrared spectroscopy

The infrared spectra of solid samples were collected using a diffuse reflectance cell (Harrick Scientific) and a IFS-66V/S spectrometer (Bruker). 300 spectra at a resolution of 4 cm^−1^ were averaged over 4000–370 cm^−1^ range.

### Thermogravimetry

Prior to burning the CA sample was subjected to UV/ozone radiation for 10 min and then was burned at 350°C in an ashing furnace (Carbolite) for 4×30 min. The gradually decreasing weight of CA was measured by using AE100 microbalances (Mettler Toledo) with an accuracy of six digits after each time interval until the weight remained constant.

## Supporting Information

Figure S1 PCR analysis of CA inclusions. Escherichia coli 16s rDNA detected in five patient specimens are shown in lines (1–5), negative control - in line (6), φX174 RF DNA marker - in line (7).
(0.14 MB JPG)

Figure S2 Congo red staining of CA. CA inclusions were stained with Congo red and observed in polarized microscope with 40-fold magnification.
(0.09 MB JPG)
