# Supplementary figures and images for: Amyloid Formation by the Pro-Inflammatory S100A8/A9 Proteins in the Ageing Prostate
Source: PLoS One. 2009 May 15;4(5):e5562. doi: 10.1371/journal.pone.0005562 (PMC2678268; doi:10.1371/journal.pone.0005562)

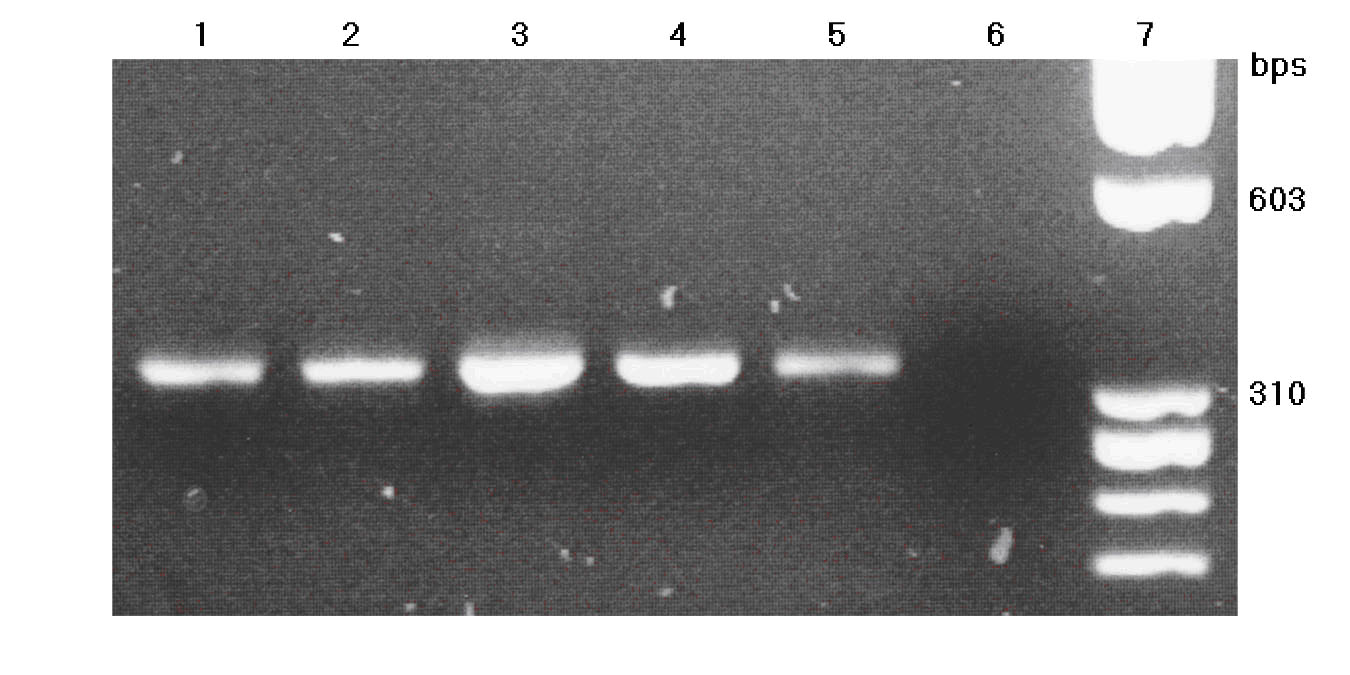

Supplement: Figure S1 — PCR analysis of CA inclusions. Escherichia coli 16s rDNA detected in five patient specimens are shown in lines (1–5), negative control - in line (6), φX174 RF DNA marker - in line (7). (0.14 MB JPG) [file pone.0005562.s001.jpg]

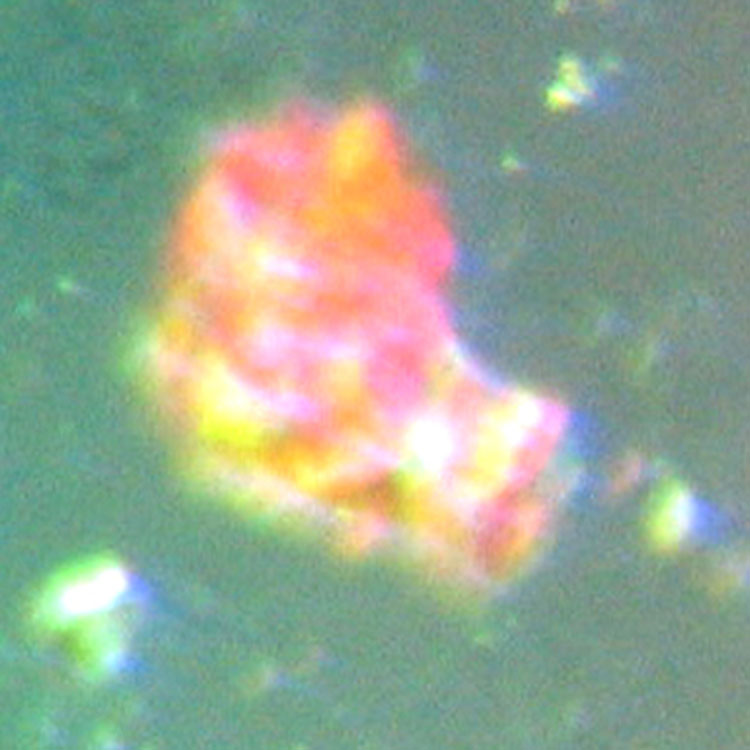

Supplement: Figure S2 — Congo red staining of CA. CA inclusions were stained with Congo red and observed in polarized microscope with 40-fold magnification. (0.09 MB JPG) [file pone.0005562.s002.jpg]
